# Editorial: Temporomandibular disorder: new directions in research and patient care

**DOI:** 10.3389/fdmed.2024.1452938

**Published:** 2024-07-10

**Authors:** John W. Kusiak, Richard Ohrbach, Alexandre Da Silva, Mildred Embree

**Affiliations:** ^1^ Retired, Santa Fe, NM, United States; ^2^ Department of Oral Diagnostic Sciences, University at Buffalo School of Dental Medicine, Buffalo, NY, United States; ^3^ Headache and Orofacial Pain Effort (H.O.P.E.) Laboratory, Department of Biologic and Materials Sciences, University of Michigan School of Dentistry, Ann Arbor, MI, United States; ^4^ Cartilage Biology and Regenerative Medicine Laboratory, College of Dental Medicine, Columbia University Irving Medical Center, New York, NY, United States

**Keywords:** temporomandibular disorder, biopsychosocial model, complex condition, diagnosis, omics

**Editorial on the Research Topic** Temporomandibular disorder: new directions in research and patient care

## 1 Introduction

The publication of Temporomandibular Disorders: Priorities for Research and Care (2020) by the National Academies of Sciences, Engineering, and Medicine (NASEM) (1) provided a framework for improving the treatment of patients with temporomandibular disorders (TMDs). Among eleven recommendations, several addressed the need for improving assessment of TMDs to advance patient care; developing science-based clinical practice guidelines and metrics for care; and strengthening basic, clinical, and population research. One paper in this special issue is pertinent to the first two recommendations which, for TMDs, are particularly critical due to the many problems in the clinical arena as described in the 2020 NASEM report. Two papers address the third of these recommendations, notably demonstrating what strong basic science can do to advance our knowledge of mechanisms underlying TMDs; the relevance of these two papers as basic science is described in a separate Perspective (2). Here, we provide further comments on how basic and clinical research may contribute to an improvement in TMD patient care.

## 2 Temporomandibular joints are part of TMDs but treatments focused only on the joint may not optimally address the needs of the patient with a TMD

Greene et al. critically describe how new technologies utilized in patient care may be misapplied if not scientifically validated and may lead to unnecessary treatment and potential harms to patients with a TMD. The authors advocate for a patient-centric approach to the diagnosis and treatment of TMDs with patient-reported problems as the driving factor for whether there is a disorder. This is in alignment with modern ideas of TMDs as complex conditions. The authors raise several concerns regarding the injudicious use of untested technologies in treating patients and suggest pathways to overcome these problems. Inherent in the content of this paper are the needed steps to remedy these patient risks and the less than satisfactory treatment of TMDs. In particular, the authors point out the failure of the dental and medical communities to approach the diagnosis and treatment of TMDs as complex conditions. Too many dentists still hold onto outmoded functional theories of pathoetiology of TMDs as the justification for use of these new technologies while neglecting a patient-centered approach to disease recognition.

The authors note that many new technologies lack scientific validation and too often rely on testimonials and anecdotal evidence as evidence of purported efficacy. This does not substitute for rigorous clinical research in validating new technology. The authors raise an overriding concern about patients with a TMD: what is considered normal jaw anatomy and function. They note that wide variation occurs in both, and that while tissues may change with time, patients can successfully adapt without overt impairment. While new technologies can measure these anatomical and functional changes with great precision, their utility or relevance in diagnosis of disease and treatment outcomes remains questionable if normal patient variation is not considered. In short, patient reports of problems should drive diagnosis and treatment vs. new technologies discovering and magnifying an abnormality into a clinically irrelevant condition.

Meanwhile, advances in understanding TMJ osteoarthritis stems from appropriate use of new technologies. Reed et al. use transgenic mice and *in vitro* systems to demonstrate that the subcellular dynamics of NG2/CSPG4, a transmembrane proteoglycan in TMJ fibrochondrocytes, is a critical player, not only in temporomandibular joint mechanical homeostasis and joint health, but also in the initiation and progression of cartilage degeneration in TMJ osteoarthritis. Their detailed molecular studies have identified a potentially new therapeutic target for treatment of osteoarthritic TMD. Mackie et al. describe how the integration of quantitative biomarkers of bone texture and superior joint space morphometry into machine-learning algorithms improved the classification of early stage TMD osteoarthritis. They stress the importance of bone microstructure in AI models for TMD osteoarthritis diagnosis and progression. They also note the need for a comprehensive set of variables, including clinical measures, in assessing the state of TMJ osteoarthritis, highlighting the point made by Greene et al. information from technology must be coupled with understanding the patient.

## 3 Additional work is needed

The underlying message from Greene et al. suggests several important additional remedies to ameliorate the health services-related problems of inappropriate introduction of new technology and its measurements. The reports by Reed et al. and Mackie et al. are excellent examples of new research directions that will assist classification and diagnosis as well as provide new targets for treatment of TMJ OA. First, new approaches in TMD research are needed; patient-centric approaches that incorporate the integration of basic, translational, and clinical research, with adequate attention to disease phenomenology, should be encouraged. For example, integrated “omics” approaches are needed to better stratify TMD patients. Combining several genomic approaches such as epigenomics and transcriptomics as well as proteomics and metabolomics together with behavioral research would allow the identification of those TMD patients who are likely to benefit from specific treatments. Bioinformatic analysis of such data is a critical component of this and its application also may point toward novel approaches to treating these patients. Treatment validation studies will require more emphasis and should in particular incorporate attention to the phenomenology of the disease. The use of new technologies in diagnosis and treatment of TMDs needs to be critically evaluated in defining treatable abnormalities and defining a new normal for jaw anatomy and function.

Second, dental education and training needs to be more scientifically rigorous. Concepts of interprofessional care and critical evaluation of scientific evidence need inclusion and emphasis in dental, medical, and other professional school curricula despite the oft-expressed disdain for this type of curricula inclusion.

And third, patient education and awareness must be improved. Detailed information about TMD diagnostics and treatments needs to become more available and disseminated. This information should address disease characteristics, patient evaluation standards, and prognostic implications; rehabilitative vs. irreversible approaches; safety of proposed treatments; and supporting scientific evidence. With this information, patients can make informed decisions with their interprofessional treatment team about their best care.
